# *Persicaria lapathifolia* Essential Oil: Chemical Constituents, Antioxidant Activity, and Allelopathic Effect on the Weed *Echinochloa colona*

**DOI:** 10.3390/plants10091798

**Published:** 2021-08-29

**Authors:** Ahmed M. Abd-ElGawad, Giuliano Bonanomi, Sarah A. Al-Rashed, Abdelsamed I. Elshamy

**Affiliations:** 1 Plant Production Department, College of Food & Agriculture Sciences, King Saud University, P.O. Box 2460, Riyadh 11451, Saudi Arabia; 2 Department of Botany, Faculty of Sciences, Mansoura University, Mansoura 35516, Egypt; 3 Department of Agricultural Sciences, University of Naples Federico II, Via Università 100, 80055 Portici, Italy; giuliano.bonanomi@unina.it; 4 Task Force on Microbiome Studies, University of Naples Federico II, 80131 Naples, Italy; 5 Department of Botany and Microbiology, College of Science, King Saud University, P.O. 2455, Riyadh 11451, Saudi Arabia; salrashed@ksu.edu.sa; 6 Chemistry of Natural Compounds Department, National Research Centre, 33 El Bohouth St., Dokki, Giza 12622, Egypt; elshamynrc@yahoo.com

**Keywords:** pale smartweed, essential oil, phytotoxicity, green chemistry, herbicides

## Abstract

The exploration of new green, ecofriendly bioactive compounds has attracted the attention of researchers and scientists worldwide to avoid the harmful effects of chemically synthesized compounds. *Persicaria lapathifolia* has been reported to have various bioactive compounds, while its essential oil (EO) has not been determined yet. The current work dealt with the first description of the chemical composition of the EO from the aerial parts of *P. lapathifolia*, along with studying its free radical scavenging activity and herbicidal effect on the weed *Echinochloa colona*. Twenty-one volatile compounds were identified via GC–MS analysis. Nonterpenoids were the main components, with a relative concentration of 58.69%, in addition to terpenoids (37.86%) and carotenoid-derived compounds (1.75%). *n*-dodecanal (22.61%), α-humulene (11.29%), 2,4-dimethylicosane (8.97%), 2*E*-hexenoic acid (8.04%), γ-nonalactone (3.51%), and limonene (3.09%) were characterized as main compounds. The extracted EO exhibited substantial allelopathic activity against the germination, seedling root, and shoot growth of the weed *E. colona* in a dose-dependent manner, showing IC_50_ values of 77.27, 60.84, and 33.80 mg L^−1^, respectively. In addition, the *P. lapathifolia* EO showed substantial antioxidant activity compared to ascorbic acid as a standard antioxidant. The EO attained IC_50_ values of 159.69 and 230.43 mg L^−1^, for DPPH and ABTS, respectively, while ascorbic acid exhibited IC_50_ values 47.49 and 56.68 mg L^−1^, respectively. The present results showed that the emergent leafy stems of aquatic plants such as *P. lapathifolia* have considerably low content of the EO, which exhibited substantial activities such as antioxidant and allelopathic activities. Further study is recommended to evaluate the effects of various environmental and climatic conditions on the production and composition of the EOs of *P. lapathifolia*.

## 1. Introduction

Essential oils (EOs) are complex mixtures of small compounds, and they have been documented to have persuasive medicinal potentialities including antioxidant [1,2], antibacterial [3,4], anti-inflammatory [5], anticancer [6], antiaging [2] antipyretic [5], and other activities. Because of these activities, EOs have been integrated into the food and cosmetics industries [7]. In the agriculture sector, EOs can be integrated as green, ecofriendly bioherbicides, where they can substitute the synthetic and chemical herbicides that are responsible for environmental pollution and affect human health [8].

Allelopathy is defined as the chemical interference between plants [9]. Understanding this biological phenomenon aids the development of applications for natural and agricultural systems [10]. Allelopathic interactions are considered to be crucial for the success of many invasive plants and are a key element in determining species distribution and abundance within plant ecosystems. Temperature, water content, salinity, nutrition availability, competitive stress, and microbiota are among the most important variables influencing allelopathy [11,12,13,14]. EOs from various wild plants have been reported as allelochemicals that interfere with the germination and growth of weeds [6,12,14,15]. These EOs have promising characteristics against weeds, as they are ecofriendly, have low resistance from weeds, and possess a wide allelopathic spectrum [8]. Over 3000 plant species have been studied for their EO composition, and hundreds of these EOs have been produced commercially [16]. In consequence, the exploration of new EOs from various wild plants with biological activities has attracted the attention of researchers and scientists worldwide.

**Figure 1:**
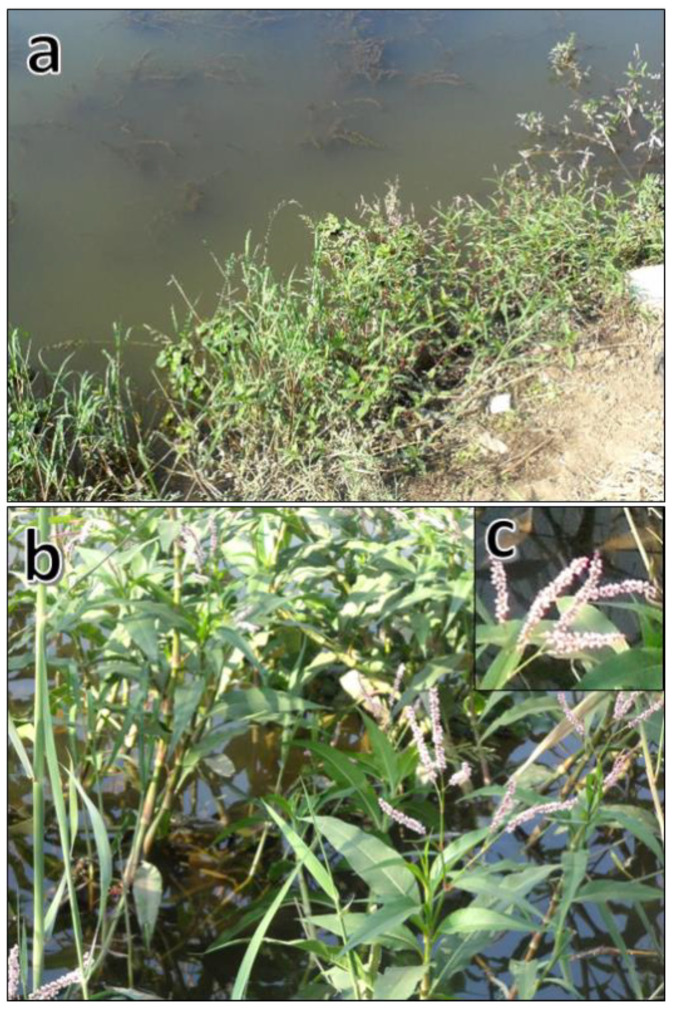
*Persicaria lapathifolia* (L.) Delarbre: (**a**) overview of the aerial parts growing on the canal bank habitat, (**b**) close view of the aerial parts, and (**c**) close view of the inflorescence.

Polygonaceae is an important edible families of flowering plants and has a worldwide distribution with 46 genera and 1100 species [17,18]. In the flora of Egypt, there are 28 species of Polygonaceae, belonging to eight genera [17]. *Persicaria* is commonly known as smartweeds or knotweeds, and this genus comprises about 150 herbaceous cosmopolitan species. Most species are found in temperate regions, with a few others are found in tropical and subtropical regions from sea level to a range of different altitudes [19]. Seven *Persicaria* species have been recorded in the flora of Egypt. *Persicaria lapathifolia* (L.) Delarbre (synonym *Polygonum lapathifolium*) is commonly known as pale persicaria, pale smartweed, or curlytop knotweed (Figure 1).

*Persicaria lapathifolia* is an annual herb that grows up to 80 cm and has reddish stems and alternate leaves [17]. This plant has grown worldwide (cosmopolitan) and inhabits various moist habitats and in agricultural fields. This species is considered a troublesome weed. *Persicaria lapathifolia* has many uses in traditional medicine such as antibacterial, antiviral, anti-inflammatory, astringent, antiseptic, anti-stomach complaint, hepatoprotective, and antifungal uses in addition to its use for the treatment of dysentery, burns, and fevers [20]. Several studies have revealed that different extracts and isolated metabolites of this plant have antimicrobial [21], anthelmintic and antiemetic [20], anticancer [22], antioxidant, α/β-glucosidase inhibitory, and anticholinesterase activity [23]. These activities were ascribed to the numerous phytoconstituents in this plant such as flavonoids, chalcones, acylated flavonoids, ferulate esters, and phenolic acids [21,22,23,24,25]. However, to our knowledge, the EO of *P. lapathifolia* has not been described yet, and in consequence, its EO’s allelopathic effect against weeds has not been studied. *Echinochloa colona* (L.) Link is commonly recognized as jungle rice because of its excellent competition with rice [26]. It is a noxious worldwide distributed weed that infests many crops (rice, maize, sugarcane, and cotton) as well as other habitats such as roadsides, gardens, disturbed sites, fallow lands, and pastures [27]. This weed is hardly controlled in the agroecosystem because of its diverse ecotypes, vast production of seeds, short seed dormancy, fast growth rate, high competitive perspective, allelopathic interference, and herbicide resistance [26].

Thereby, the present study aimed to analyze for the first time the chemical composition of the volatile oil extracted from the aerial parts of *P. lapathifolia*, evaluate its allelopathic activity of the EO on the weed *E. colona*, and determine its antioxidant activity.

## 2. Results and Discussion

### 2.1. Chemical Composition of P. lapathifolia EO

The hydrodistillation of the aerial parts of *P. lapathifolia* via the Clevenger apparatus produced 0.18% (*v*/*w*) of a dark yellow oil accompanied by a slight scent. This yield was lower than those reported from other *Persicaria* species such as Vietnamese *P. odorata* (0.41%) [28], Malaysian *P. odorata* (0.64%) [29], and *P. hydropiper* (0.70%) [15]. Overall, twenty-one volatile components were identified via GC–MS analysis, representing 98.3% of the total mass of oil (Table 1).

Eight classes of the compounds were characterized for the EO, including monoterpenes (hydrocarbons and oxygenated), sesquiterpenes (hydrocarbons and oxygenated), diterpenes (hydrocarbons and oxygenated), carotenoid-derived compounds, and other nonterpenoid compounds (Table 1). Among these components, nonterpenoid constituents represented the predominant type of compounds, with a relative concentration of 58.69%. This result is consistent with the previously described essential oils from Vietnamese [28] and Australian [30] *P. odorata*. In contrast, Dũng, et al. [31] described that sesquiterpene hydrocarbons were the main compounds of the Vietnamese *P. odorata* along with the oxygenated constituents. Herein, 11 nonterpenoids were identified, comprising *n*-dodecanal (22.61%), 2,4-dimethylicosane (8.97%), 2*E*-hexenoic acid (8.04%), and γ-nonalactone (3.51%) as the abundant compounds, while *n*-tricosane (1.18%) was determined as a minor compound. The preponderance of the alkyl aldehyde, *n*-dodecanal, in the EO of *P. lapathifolia* is in harmony with the published data on the EOs of Vietnamese and Australian *P. odorata* [28,30].

Monoterpenes were determined with a relative concentration of 17.23% of the EO mass. They can be divided into monoterpene hydrocarbons (6.80%) and oxygenated monoterpenes (10.43%). Three monoterpene hydrocarbon compounds were assigned, of which limonene (3.09%) was the major and α-pinene was a minor compound. Limonene is not a widely distributed compound in the EOs of *Persicaria* plants, although it has been reported as a major constituent of the EOs of several species such as *Schinus terebinthifolius* [32], *Callistemon viminalis* [33], *Artemisia scoparia* [34], *Heterothalamus psiadioides* [35], and *Carum carvi* [36].

On the other side, 2-methyl butyl isovalerate was the only identified oxygenated monoterpene, with a relative concentration of 10.43%. Isovalerate derivatives have been described in the EOs of several plants such as *Eucalyptus brockwayii* [37], Algerian *Daucus gracilis* [38], and *Chamaemelum fuscatum* [39].

Sesquiterpenes made up a relative concentration of 15.77% of the total oil mass. They consisted of sesquiterpene hydrocarbons (12.58%) and oxygenated sesquiterpene (3.19%). α-Humulene (11.29%), and *trans*-caryophyllene (1.29%) were the only identified sesquiterpene hydrocarbons. The two compounds are popular in the EOs of the plants belonging to *Persicaria* such as Vietnamese *P. odorata* [28,31] and Australian *P. odorata* [30]. In the EO of *P. lapathifolia*, diterpenes represented 4.86% of the total oil mass, including one diterpene hydrocarbon, phytane (3.78%), and one oxygenated compound, abienol (1.08%). Diterpenes have been recorded in the EOs of *Persicaria* species as traces [28,30,31]. The scarcity of diterpenoids in EOs derived from plants is a common phenomenon with a few exceptions such as *Araucaria heterophylla* [5] and *Calotropis procera* [14].

Carotenoid-derived components were represented by only the common compound, hexahydrofarnesyl acetone (1.75%), which has been characterized in the EOs of many plants such as *Launaea mucronata*, *Launaea nudicaulis* [40], and *Heliotropium curassavicum* [13].

### 2.2. Allelopathic Activity of P. lapathifolia EO

**Figure 2:**
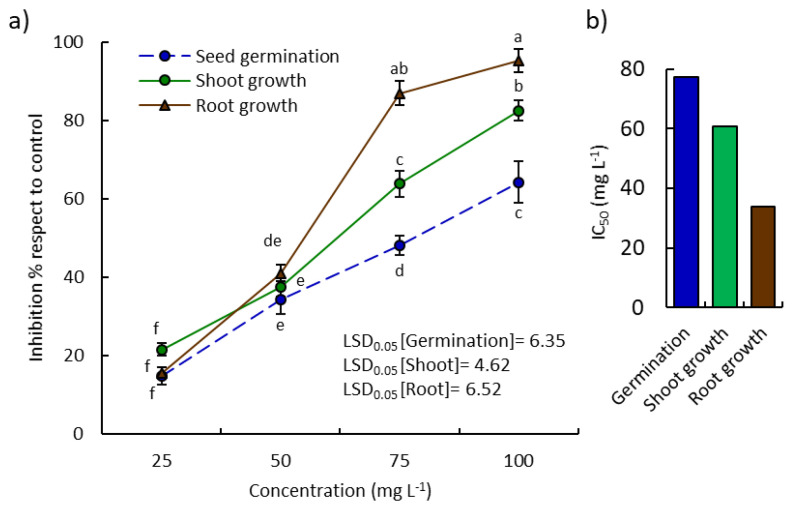
Allelopathic activity of the *Persicaria lapathifolia* essential oil. (**a**) Inhibitory effect on the germination, seedling root, and shoot growth of the weed *Echinochloa colona*; (**b**) IC_50_ values. Different letters mean a significant difference in values after Tukey’s HSD test (*p* < 0.05).

The extracted EO of *P. lapathifolia* exhibited substantial allelopathic activity against the germination, seedling root, and shoot growth of the weed *E. colona* in a dose-dependent manner (Figure 2). At the highest concentration (100 mg L^−1^), the germination, seedling root, and shoot growth were inhibited by 64.25, 82.48, and 95.25%, respectively. Additionally, the EO showed IC_50_ values of 77.27, 60.84, and 33.80 mg L^−1^, respectively (Figure 2). It is clear that the seedling growth was more inhibited than germination. However, the root growth of *E. colona* was more sensitive to the EO than the shoots, and this observation was in harmony with other studies [13,41]. This could be attributed to the permeability of the root cells and the direct contact with the medium that contained the EO [14,41,42].

To our knowledge, the allelopathic activity of *P. lapathifolia* EO has not been described yet. Herein, the EO of *P. lapathifolia* was found to exhibit allelopathic effects on *E. colona*. Many published data have revealed the principal and direct relationship between the allelopathic properties of EOs derived from plants and their chemical compositions [4,14,40,41,42]. Therefore, the observed allelopathic activity of *P. lapathifolia* EO might be ascribed to its chemical constituents, especially the main compounds *n*-dodecanal, α-humulene, 2,4-dimethylicosane, and 2*E*-hexenoic acid, γ-nonalactone, and limonene. These compounds could act either singularly or in a synergistic manner as allelochemicals that inhibit germination and seedling growth [41]. The allelopathic effect of allelochemicals may occur by inhibition of cell division, reduction of respiration, affecting photosynthesis, inhibition of enzymatic systems, affecting nucleic acid, or induction of reactive oxygen species in plant cells [43,44,45]. Humulene has been reported as a major compound in the EO of *Symphyotrichum squamatum*, which exhibited significant allelopathic activity on the weed *Bidens pilosa* [12]. Also, limonene has been reported as a major compound in the EOs of various plants that exhibited considerable allelopathic activity such as *Heterothalamus psiadioides* [35], *Agastache rugosa* [46], *Schinus terebinthifolius* [32], *Callistemon viminalis* [33], *Artemisia scoparia* [34], *Pinus pinea* [47], and *Carum carvi* [36].

It is worth mentioning here that *E. colona* has been reported to have allelopathic activity against various crops and weeds such as rice, soybean [48], and *Avena fatua* [49]. Also, the allelopathic activity of various plant extracts, such as those of *Sorghum bicolor*, *Helianthus annuus*, *Brassica campestris* [50], *Mikania micrantha*, *Clidemia hirta*, *Dicranopteris linearis*, and *Ageratum conyzoides* [51], has been studied against *E. colona*. However, no study has revealed the activity of the essential oil of *P. lapathifolia* against this weed. In this line, the present study showed that the EO of the aerial parts of *P. lapathifolia* could be used in the management of weeds as a green, ecofriendly herbicide, particularly against species of *Echinochloa*, including *E. colona*, which has been reported to be resistant against herbicides [52].

### 2.3. Antioxidant Activity of P. lapathifolia EO

**Figure 3:**
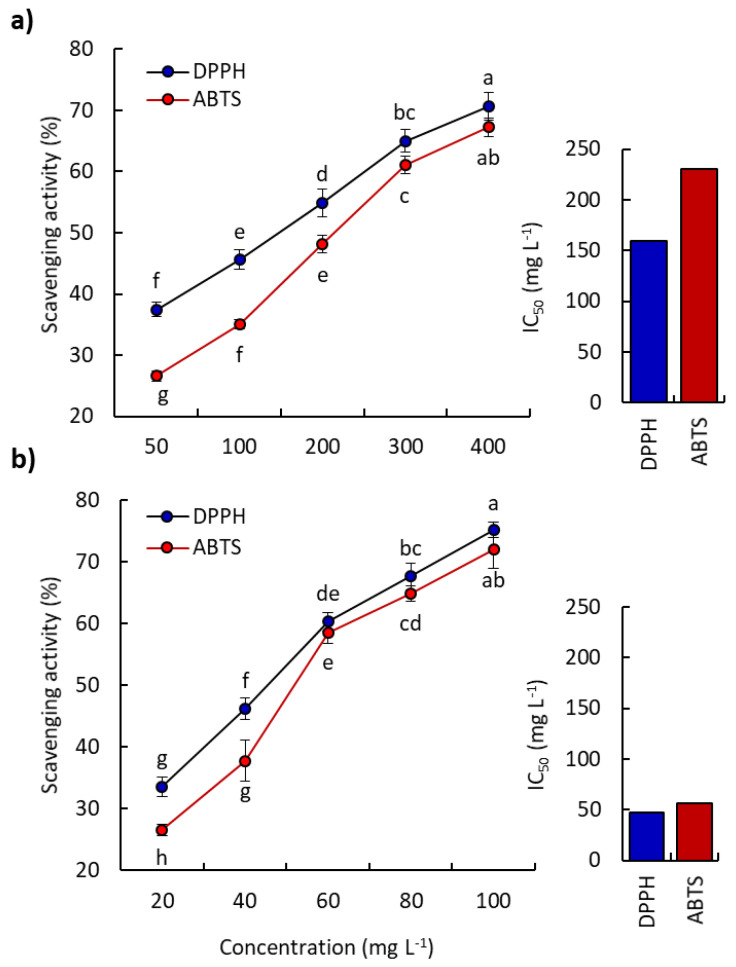
Antioxidant activity of *Persicaria lapathifolia* essential oil (**a**) and ascorbic acid as a standard antioxidant (**b**). Different letters mean a significant difference in values after Tukey’s HSD test (*p* < 0.05).

The EO of *P. lapathifolia* was tested for antioxidant activity via the reduction of the free radicals DPPH and ABTS, and it showed a substantial antioxidant activity compared to ascorbic acid as a standard antioxidant (Figure 3). By increasing the concentration of the EO, the reduction of radicals was increased. At the highest concentration of the EO (400 mg L^−1^), the DPPH and ABTS were reduced by 70.68 and 67.23%, respectively. The EO showed IC_50_ values of 159.69 and 230.43 mg L^−1^, respectively (Figure 3a), while ascorbic acid exhibited IC_50_ values 47.49 and 56.68 mg L^−1^, respectively (Figure 3b).

The antioxidant activity of the EO is usually correlated to the oxygenated compounds in the EO profile [8,12,41]. Thereby, the substantial antioxidant activity of EO of *P. lapathifolia* could be attributed to its high content of oxygenated components (> 70%), especially oxygenated hydrocarbons. Additionally, terpenoid compounds have been stated to have important functions as free radical scavengers, especially the oxygenated derivatives [12,40]. In the present study, about 38% of the EO mass consisted of terpenoids, including 15% oxygenated derivatives that could be responsible for the scavenging of free radicals. Many EOs of plants have been proven to exhibit significant antioxidant effects via different methods because of their high percentages of terpenoids, such as those of *Euphorbia mauritanica* [1], *Deverra tortuosa* [3], and *Launaea* species [40].

## 3. Materials and Methods

### 3.1. Plant Materials Collected and Preparation

The aerial parts of *Persicaria lapathifolia* were collected in May from a population growing on the canal bank habitat of an irrigation canal near the city of Mansoura, Egypt (31.0708553N 31.4394701E). The collected aerial parts were air dried at room temperature (25 ± 3 °C), crushed gently by hand, and packed in a paper bag till further analyses. A plant voucher specimen was deposited in the herbarium of the Department of Botany, Faculty of Science, Mansoura University, Egypt.

### 3.2. Extraction of EO, GC-MS Analysis, and Identification of Chemical Constituents

About 250 g of the air-dried powder aerial parts of *P. lapathifolia* was subjected to hydrodistillation over Clevenger-type apparatus for three hours, and the dark yellow, oily layer was separated by n-hexane via a separating funnel. The EO chemical composition was analyzed and identified based on gas chromatography–mass spectrometry (GC–MS) using the instrument at the Medicinal and Aromatic Plants Research Dept., National Research Center, Egypt. The instrument comprises a TRACE GC Ultra Gas Chromatographs (THERMO Scientific Corp., Miami, CA, USA) and a thermo mass spectrometer detector (ISQ Single Quadrupole Mass Spectrometer; Model ISQ spectrometer, THERMO Scientific Corp., Miami, CA, USA). The system was equipped with a TR-5 MS column with specifications of 30 m × 0.32 mm i.d. and 0.25 µm film thickness. Helium was used as a carrier gas at a flow rate of 1.0 mL min^−1^ and a split ratio of 1:10. The temperature program started at 60 °C for one minute and was then raised 4.0 °C every minute to 240 °C, then held for one minute. Electron ionization (EI) at 70 eV with a spectral range of *m*/*z* 40–450 was used for performing mass spectra. The chemical constituents of the EO were tentatively identified by their retention indices (relative to n-alkanes C_8_-C_22_) and mass spectrum matching to the Wiley spectral library collection and the NSIT library database [41].

### 3.3. Allelopathic Bioassay

The extracted EO from the aerial parts of *P. lapathifolia* was tested in vitro for its allelopathic activity against the germination and seedling growth of the weed *E. colona*. The ripened seeds of *E. colona* were collected from a cultivated field near the city of Manzala, Al-Dakahlya Governorate, Egypt (31.1691466N 31.896379E). The homogenized seeds in size and color were sterilized with 0.3% NaClO and dried in a sterilized condition. The viability of seeds was preliminarily tested and found to be 94.56% ± 3.25. For bioassay, different concentrations (0, 25, 50, 75, and 100 mg L^−1^) of the extracted EO were prepared in 1% Tween^®^ 80 (Sigma-Aldrich, Darmstadt, Germany) as an emulsifier. Twenty sterilized seeds were arranged over a filter paper (Whatman No. 1) in Petri dishes. About four mL of each concentration and Tween^®^ 80 (as control) were poured over the filter paper, and the dishes were sealed with Parafilm^®^ tape (Sigma, St. Louis, MO, USA) to avoid the loss of EO [41]. For each concentration, five dishes were tested, and the experiment was repeated three times. A total of 75 dishes (5 treatments (4 concentrations + control) × 5 dishes (replications) × 3 times) were prepared and incubated at 27 °C in a growth chamber with adjusted light conditions of 16 h light and 8 h dark. After ten days of incubation, the number of germinated *E. colona* seeds was counted, and the lengths of the seedling root and shoot of the weed were measured. The inhibition of germination and growth was calculated with respect to control according to the following equation:

$$\mathrm{Inhibition}\;\left(\%\right)=100\times \frac{\left({\frac{N_{control}}{L}}_{control}-{\frac{N_{treatment}}{L}}_{treatment}\right)}{{\frac{N_{treatment}}{L}}_{control}}$$

where *N* is the number of germinated seeds and *L* is the length of the seedling root or shoot.

### 3.4. Antioxidant Activity Estimation

The antioxidant activity of the extracted EO was assessed by its ability to reduce the free radicals 2,2-diphenyl-1-picrylhydrazyl (DPPH, Sigma-Aldrich, Germany) and 2,2′-azinobis(3-ethylbenzothiazoline-6-sulfonic acid) (ABTS, Sigma-Aldrich, Germany). According to Miguel [53], a range of concentrations of the EO (50–400 mg L^−1^) were prepared in MeOH. This range was selected based on the observed scavenging percentage, i.e., to be suitable to determine the IC_50_ (the concentration of EO necessary to scavenge the radical by 50%) [3]. In glass tubes, 2 mL of each concentration was mixed vigorously with 2 mL of freshly prepared 0.3 mM DPPH. Negative control was performed using MeOH treated with DPPH-like treatment. The reaction mixtures were kept in dark conditions at room temperature for 30 min, and the absorbance was measured immediately at 517 nm by spectrophotometer (Spectronic 21D model).

For confirmation, the scavenging of ABTS was performed following the method of Re et al. [54]. Briefly, the ABTS radical was prepared by mixing about 7 mM ABTS (1/1, *v*/*v*) with 2.45 mM potassium persulfate, and this mixture was stored in dark conditions at 25 ± 2 °C for 16 h. In glass tubes, 2 mL of the prepared ABTS radical and 0.2 mL of each EO concentration (50–400 mg L^−1^) were mixed well and kept for 6 min at room temperature. The absorbance was measured by a spectrophotometer at 734 nm. Moreover, the antioxidant activity of ascorbic acid as a standard antioxidant was determined following the same procedures for DPPH and ABTS using a range of 20–100 mg L^−1^. The scavenging activity was calculated according to the following equation:

$$Radical\;scavenging\;activity\;\left(\%\right)=100\times \left[1-\left(Absorbance_{sample}/Absorbance_{control}\right)\right]$$

### 3.5. Statistical Analysis

The experiment of allelopathic activity was performed three times with five replicas per treatment. The mean values and standard error were calculated, while the raw data were subjected to one-way ANOVA followed by Tukey’s HSD test. Moreover, the data of antioxidant activity of the EO and ascorbic acid with three replicates were subjected to one-way ANOVA followed by Tukey’s HSD test as well. The analysis was accomplished in the CoStat program (version 6.311, CoHort Software, Monterey, CA, USA). The IC_50_ for allelopathic and antioxidant assays were calculated graphically using MS Excel.

## 4. Conclusions

The current study showed for the first time the chemical composition of the EO from the aerial parts of *P. lapathifolia*. The EO had 21 compounds, with 58.69% as nonterpenoids and 37.86% as terpenoids. The main compounds were *n*-dodecanal, α-humulene, 2,4-dimethylicosane, 2*E*-hexenoic acid, γ-nonalactone, and limonene. The EO of *P. lapathifolia* showed substantial herbicidal activity against the weed *E. colona*; 100 mg L^−1^ of this EO inhibited the germination, seedling root, and shoot growth by 64.25, 82.48, and 95.25%, respectively. Also, the EO exhibited considerable antioxidant activity compared to ascorbic acid as a standard. The present results showed that the emergent leafy stems of aquatic plants such as *P. lapathifolia* have considerably low content of the EO, which exhibited substantial activities such as antioxidant and allelopathic activities. Further study is recommended to evaluate the effects of various environmental and climatic conditions on the production and composition of the EOs of *P. lapathifolia*.

## Figures and Tables

**Table 1:** Essential oil chemical composition from aerial parts of *Persicaria lapathifolia*.

No	Rt ^a^	Relative Conc. (%) ^b^	Compound Name	Type	Identification ^c^	
					KI_Observed_ ^d^	KI_Published_ ^e^
1	3.23	1.74 ± 0.02	α-Pinene	MH	932	935
2	4.14	1.97 ± 0.05	Sabinene	MH	969	966
3	5.43	8.04 ± 0.06	2*E*-Hexenoic acid	Others	1007	1009
4	13.84	3.09 ± 0.05	Limonene	MH	1024	1028
5	14.54	10.43 ± 0.14	2-Methyl butyl isovalerate	OM	1104	1104
6	18.26	1.98 ± 0.03	Hydrocinnamyl alcohol	Others	1227	1232
7	20.12	3.51 ± 0.06	γ-Nonalactone	Others	1361	1367
8	21.67	1.29 ± 0.02	*trans*-Caryophyllene	SH	1408	1403
9	22.64	22.61 ± 0.16	*n*-Dodecanal	Others	1408	1415
10	23.07	11.29 ± 0.13	α-Humulene	SH	1452	1459
11	32.46	2.69 ± 0.04	2-Ethyl chromone	Others	1614	1618
12	32.84	3.78 ± 0.04	Phytane	DH	1810	1818
13	36.88	1.75 ± 0.02	Hexahydrofarnesyl acetone	Car	1845	1847
14	38.16	3.19 ± 0.03	Carissone	OS	1927	1934
15	39.42	8.97 ± 0.08	2,4-Dimethylicosane	Others	2080	2087
16	40.02	1.08 ± 0.04	Abienol	OD	2150	2156
17	42.56	2.27 ± 0.06	*n*-Docosane	Others	2200	2202
18	46.02	1.18 ± 0.03	*n*-Tricosane	Others	2300	2300
19	46.2	2.72 ± 0.05	*n*-Nonacosane	Others	2900	2905
20	49.22	3.01 ± 0.04	*n*-Hentriacontane	Others	3100	3101
21	51.14	1.71 ± 0.03	*n*-Dotriacontane	Others	3200	3203
		6.80 ± 0.07	Monoterpene Hydrocarbons (MH)			
		10.43 ± 0.14	Oxygenated Monoterpenes (OM)			
		12.58 ± 0.12	Sesquiterpene Hydrocarbons (SH)			
		3.19 ± 0.03	Oxygenated Sesquiterpenes (OS)			
		3.78 ± 0.04	Diterpene Hydrocarbons (DH)			
		1.08 ± 0.04	Oxygenated Diterpenes (OD)			
		1.75 ± 0.02	Carotenoid derived compounds (Car)			
		58.69 ± 0.13	Other compounds (Others)			
		98.3	Total			

^a^ Rt: retention time, ^b^ average value ± standard deviation, ^c^ the identification of EO constituents was based on the comparison of mass spectral data and Kovats indices (KI) with those of the NIST Mass Spectral Library (2011) and Wiley Registry of Mass Spectral Data 8th edition and literature, ^d^ KI_published_: reported Kovats retention indices; ^e^ KI_Observed_: experimentally calculated Kovats index relative to C_8_–C_28_ *n*-alkanes.

## Data Availability

Not applicable.

## Sample Availability

Samples of the compounds are not available from the authors.
